# Comparing birth experiences and satisfaction with midwifery care before and after the implementation of Canada’s first Alongside Midwifery Unit (AMU)

**DOI:** 10.1371/journal.pone.0306916

**Published:** 2024-08-21

**Authors:** Beth Murray-Davis, Lindsay N. Grenier, Jenifer Li, Anne M. Malott, Cristina A. Mattison, Carol Cameron, Eileen K. Hutton, Elizabeth K. Darling

**Affiliations:** 1 Department of Obstetrics and Gynecology, McMaster Midwifery Research Centre, McMaster University, Hamilton, Ontario, Canada; 2 Department of Health Research Methods, Evidence and Impact, McMaster University, Hamilton, Ontario, Canada; 3 Markham Stouffville Hospital Alongside Midwifery Unit, Markham, Ontario, Canada; Centre of Postgraduate Medical Education, POLAND

## Abstract

**Background:**

Globally, midwifery-led birthing units are associated with favourable clinical outcomes and positive birth experiences. As part of our evaluation of Canada’s first Alongside Midwifery Unit (AMU) at Markham Stouffville Hospital, we sought to explore and compare birth experiences and satisfaction among midwifery clients who gave birth on the AMU with midwifery clients who gave birth on the traditional obstetric unit prior to AMU implementation.

**Methods:**

We conducted a structured, online, cross-sectional survey of midwifery clients in the six months before, and up to 18 months after, opening of the AMU at Markham Stouffville Hospital, Ontario Canada. The survey contained validated measures of satisfaction including personal capacity and participation; perceived safety, control, and security; professional support; and satisfaction. Descriptive statistics and tests of significance were completed in SPSS.

**Results:**

A total of 193 responses were included in our analyses (pre-AMU n = 47, post-AMU n = 146). All participants had positive experiences in the four domains assessed. Compared to those who gave birth with midwives on the Labour unit, those who gave birth on the AMU indicated more positive experiences for some measures. Perceptions pertaining to being an active participant in care, to security and sense of control were more positive among those who gave birth on the AMU.

**Conclusion:**

The AMU in Ontario is associated with high levels of satisfaction during birth, particularly the perception of being actively engaged in decision making, having a sense of control and safety, and having confidence in the care provider team. Care received on the AMU does not compromise birth experiences or satisfaction and may be associated with greater autonomy and agency for the person giving birth.

## Introduction

In Canada, pregnant people can choose between family physicians, midwives, or obstetricians as their primary care provider for pregnancy and birth. Demand for midwifery in Canada has been increasing over the past 20 years [1]. Currently, midwives are the primary care provider for one in ten births across the country and one in five births in Ontario [2]. Midwives provides comprehensive perinatal care from early pregnancy, during birth and up to six weeks postpartum, that centres the principles of continuity of care, informed choice, and choice of birthplace [3,4]. Midwifery care is publicly funded by the government. Midwives work in group practices providing continuity of care to a caseload of clients. They work in independent community practice offices with access to hospital privileges to attend their clients during hospital birth. Although midwives offer clients a choice of birthplace including home, hospital and where available, birth centre, the majority of midwife-attended births in Ontario occur within a hospital setting on the Labour Unit [5]. In Canada, midwifery care has been demonstrated to reduce perinatal interventions [6,7] and is associated with high rates of satisfaction compared to obstetrician-led care [8].

In 2018, the Ministry of Health in Ontario funded a pilot to provide an alternative model of intrapartum care for midwifery clients to give birth at an alongside midwifery unit (AMU) at Markham Stouffville Hospital (MSH). The AMU is located within the hospital and operates separately from, but adjacent to, the obstetric unit. The proximity to the obstetric unit allows for a smooth transfer of care, if needed [9]. Community-based primary midwives provide routine care for their clients throughout the prenatal and postpartum periods and attend births on the AMU with a hospitalist midwife in the role of the second midwife who provides infant care at the time of birth. A hospitalist midwife fields early labours calls, conducts triage assessments, facilitates admission for clients, provides relief for the primary midwife, supports the primary midwife during birth, and acts as a resource for midwives and learners throughout pregnancy, postpartum, and intrapartum periods. Although it is the first of its kind in Canada, midwife-led units (MLUs) have previously been implemented in countries where midwifery services are well integrated such as, the United Kingdom [10], New Zealand [11], the Netherlands [12], France [13], and Belgium [14]. Previous research has found that people with low-risk pregnancies who give birth in MLUs experience fewer maternal complications and birth interventions—such as induction of labour, epidural analgesia, instrumental birth, perineal lacerations, and birth via caesarean section [11,13–16].

Satisfaction continues to be an important indicator of quality of care within the healthcare system [17] and has been demonstrated to impact the health and well-being of the birthing person and their baby [18,19]. Dissatisfaction with one’s birth-experience has been associated with negative feelings during infant feeding and bonding, increased risk of postpartum depression and post-traumatic stress disorders, and preference for caesarean delivery in future pregnancies [20]. Overall, midwifery clients in Canada are more satisfied with their birth experience compared to those who have given birth with other providers [19,21]. However, due to the novelty of the AMU in Canada, little is known about how this model of intrapartum care may impact birth experiences and satisfaction from the perspectives of midwifery clients. The purpose of the study was to measure and describe the experiences of those who gave birth on the AMU, including understanding the elements that impacted their experience and satisfaction.

## Methods

**Fig 1:**
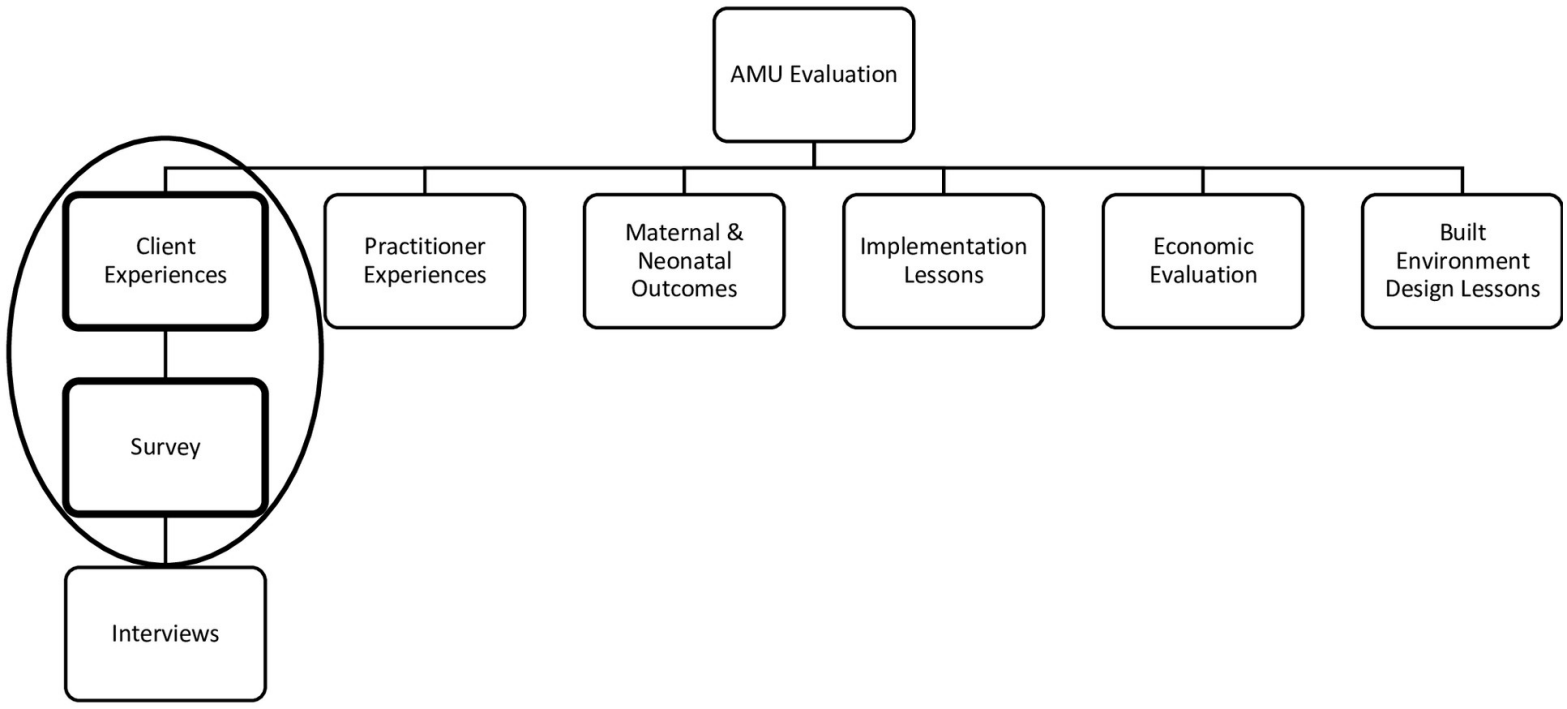
AMU evaluation study components.

To assess overall birth experience and satisfaction within the AMU, we conducted an exploratory mixed-methods study using semi-structured surveys and interviews of midwifery clients who received care at the AMU, including those whose care was transferred to an obstetrician during the intrapartum period. The results from the in-depth interviews have been reported separately [22]. In this manuscript we report the quantitative findings from structured questions from surveys. This research is part of a larger evaluation study of the AMU (Fig 1). Ethics approval was obtained from the Hamilton Integrated Research Ethics Board (HIREB study #5050, 5056) and the Markham Stouffville Hospital Research Ethics Board (study #78–1808).

We used a cross-sectional survey method to compare client satisfaction at two different birth settings for clients under midwifery care at the Markham Stouffville Hospital. Clients who were planning home births were excluded from the study. Prior to the opening of the AMU, all midwifery clients gave birth on the Labour Unit. Following the opening of the AMU, all midwifery clients began labour on the AMU. The population of midwifery clients remained unchanged, but all that changed was the physical unit where they would labour. Participants were recruited at three separate time intervals: 1) six weeks preceding the opening of the AMU (May-July 2018) (Pre-AMU), 2) within the first six months of the AMU receiving clients (Post-AMU Phase 1: September 2018-March 2019), and subsequently 3) between twelve and eighteen months (Post-AMU Phase 2: September 2018-March 2020). All midwifery clients who gave birth during these time periods at Markham Stouffville Hospital were eligible to participate. After the opening of the AMU, all midwifery clients who received care on the unit were eligible to participate, regardless of whether they were transferred to the obstetric unit during their labour.

The structured survey questions were validated measures of satisfaction and outcome reported measures from the Childbirth Experience Questionnaire (CEQ) [23] and the Canadian Maternity Experiences Survey (CMES) [24]. The survey was divided into four sections: demographics, the (CEQ), (CMES), and open-ended satisfaction questions. Survey administration and data collection was facilitated using REDCap, a secure web-based application.

Recruitment posters were distributed in participating midwifery clinics and the AMU. Pre-AMU participants completed the full online survey at their first postpartum appointment at their midwifery clinic on a tablet. Post-AMU participants were recruited prior to discharge from hospital, when they were asked to complete two questions about satisfaction and indicate whether they consented to be contacted for the full survey, also completed on a tablet. At two weeks postpartum they were emailed the full online survey. The online survey sent to post-AMU participants included three additional open-ended questions to explore their birth experience in more detail.

All participants were provided information about the study purpose, investigator, survey length, privacy, and the voluntary nature of participation. Informed consent was obtained electronically prior to beginning the survey. Participants were given the opportunity to review their answers prior to submission. All participants were offered a $20 gift card in recognition of their time and effort in participating.

Descriptive and summary statistics were used to summarize participant characteristics. Completion of the entire survey was not mandatory for inclusion in analyses. Participants with missing responses remained in the dataset. Questions with missing responses were calculated and reported based on the total number of responses, rather than the total number of participants. To assess differences between groups, independent samples t-tests and Pearson chi-square tests were conducted for continuous and nominal outcomes, respectively. All analyses were completed in SPSS (version 26).

## Results

### Participant characteristics

A total of 597 births occurred during the study period (Pre-AMU n = 84, post-AMU n = 513) and a total of 193 responses (Pre-AMU n = 47, post-AMU n = 146) were included in analyses. This yielded an overall response rate of 32.3% and provides a margin of error of 5.81% for our analyses. Overall completion and completeness rates were high (89.3% and 98.8%, respectively).

The demographic characteristics of participants are shown in Table 1. The majority (70.3%) of clients who participated in the survey were between the ages of 25 and 35 years. A statistically significant higher proportion of clients aged 30–34 years gave birth prior to the implementation of the AMU (p = 0.03). Parity was similar among clients before and after the implementation of the AMU. Education level was high across the cohort, with 90.2% of clients having received post-secondary degrees or diplomas (63.2% at a bachelor’s degree level or higher). Most participants (64.9%) reported their household income to be greater than $75,000.

**Table 1:** Participant characteristics (Pre-AMU, Post-AMU Phase 1, Post-AMU Phase 2).

Participant Characteristics	Pre-AMU (n = 47)	Post-AMU Phase 1 (n = 87)	Post-AMU Phase 2 (n = 59)	Total (n = 193)
	n (%)	n (%)	n (%)	n (%)
**Parental Age (years)**	n = 47	n = 86	n = 59	n = 192
<24	3 (6.4)	2 (2.3)	2 (3.4)	7 (3.6)
25–34	33 (70.2)	66 (56.7	36 (61)	135 (70.3)
>35	11 (23.4)	18 (20.9)	21 (35.6)	50 (26.0)
**Parity**	n = 43	n = 87	n = 58	n = 190
Nulliparous	23 (53.4)	52 (59.8)	25 (43.1)	100 (52.6)
Multiparous	20 (46.4)	34 (39.1)	33 (56.8)	87 (45.8)
**Education**	n = 47	n = 87	n = 59	n = 193
No certificate/diploma/degree	1 (2.1)	0 (0)	0 (0)	1 (0.5)
High School Diploma or equivalent	3 (6.4)	7 (8.0)	4 (6.8)	14 (7.3)
College or other non-university certificate/ diploma	15 (31.9)	16 (18.4)	15 (25.4)	46 (23.9)
University Bachelor Level	1 (2.1)	6 (6.9)	2 (3.4)	9 (4.7)
University above Bachelor Level	27 (57.4)	58 (66.7)	37 (62.7)	122 (63.2)
**Household Income**	n = 47	n = 85	n = 59	n = 191
Less than $50,000	2 (4.3)	9 (10.6)	4 (6.8)	15 (7.9)
$50,000 to $74,999	3 (6.4)	15 (17.6)	11 (18.6)	29 (15.2)
$75,000 to $99,999	9 (19.1)	19 (22.4)	8 (13.6)	36 (18.8)
over $100,000	26 (55.3)	33 (38.8)	29 (49.2)	88 (46.1)
Prefer not to answer	7 (14.9)	9 (10.6)	7 (11.9)	23 (12.0)
**Location of birth and Care provider**	-	n = 86	n = 59	n = 145
AMU, with a Midwife	-	75 (87.2)	55 (93.2)	130 (89.7)
AMU, with an OB	-	5 (5.8)	3 (5.1)	8 (5.5)
Labour Unit	-	6 (7.0)	1 (1.7)	7 (4.8)

Data is presented as count (%) unless otherwise stated. Totals may not add to 100% due to rounding.

The results are presented as four domains of the birth experience, adapted from the CEQ: Capacity and participation; Perceived safety, control, and security; Professional support; and Satisfaction [23].

### Capacity & participation

**Fig 2:**
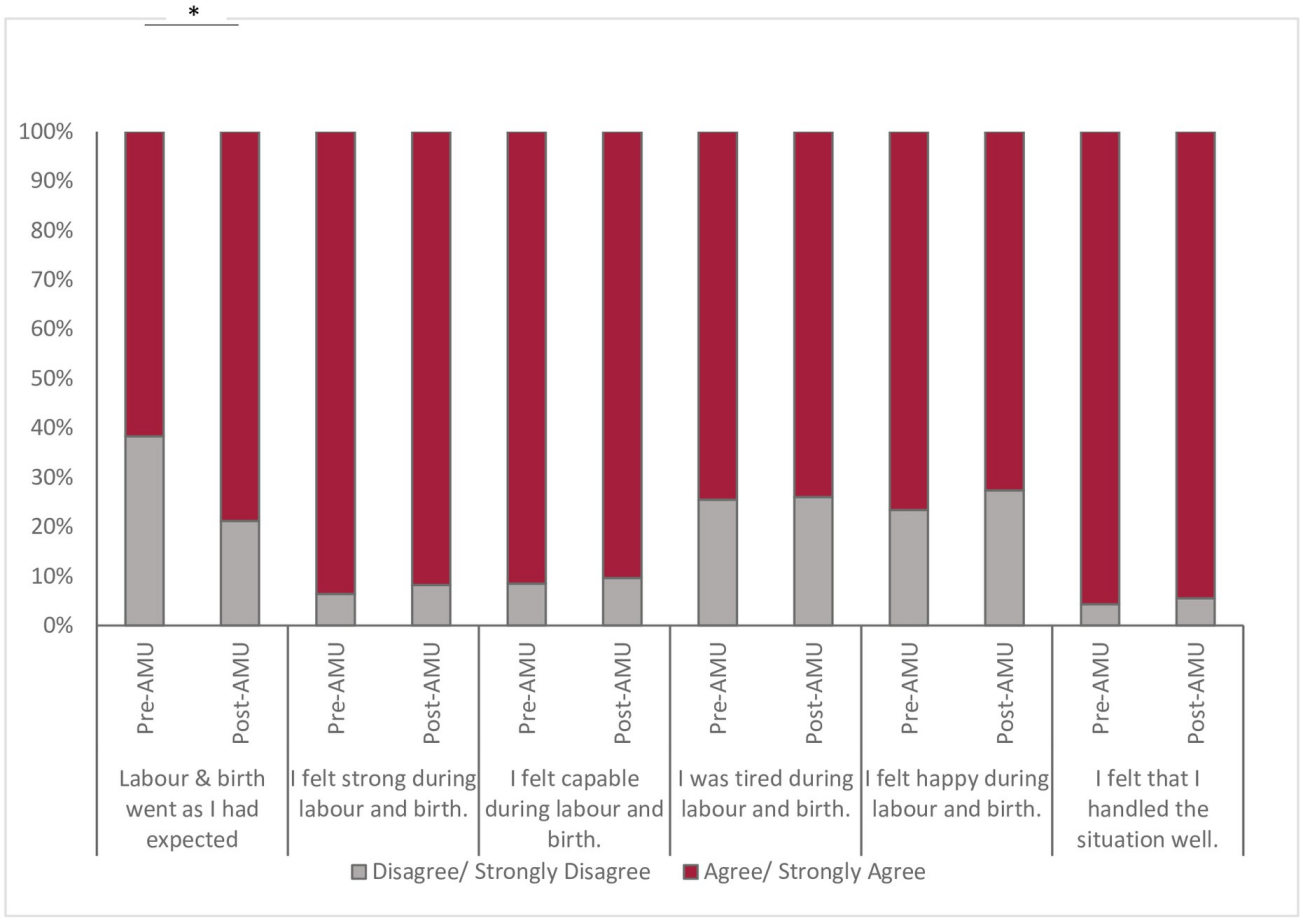
Participants’ perception of own capacity during labour and birth, pre- and post-AMU implementation. Chi-square test (*p<0.05).

Participants answered questions related to their self-efficacy, feelings, and abilities (*capacity*) during labour and birth (Fig 2). Although most clients surveyed agreed that labour went as expected (74.6%), there were higher levels of agreement among those who birthed after the implementation of the AMU (79% (95% CI 0.72,0.85) vs. 62% (95% CI 0.47,0.75, p = 0.02,) and even higher among participants who birthed 12–18 months after opening of the AMU compared to initially after opening (90% vs. 71%). Clients felt strong and capable throughout the intrapartum period and 94.8% agreed that they handled the situation well. Not surprisingly, 74% of all clients reported feeling tired during labour and birth, however feelings of joy were evident throughout their experience, as 74% agreed that they were happy during labour and birth.

**Fig 3:**
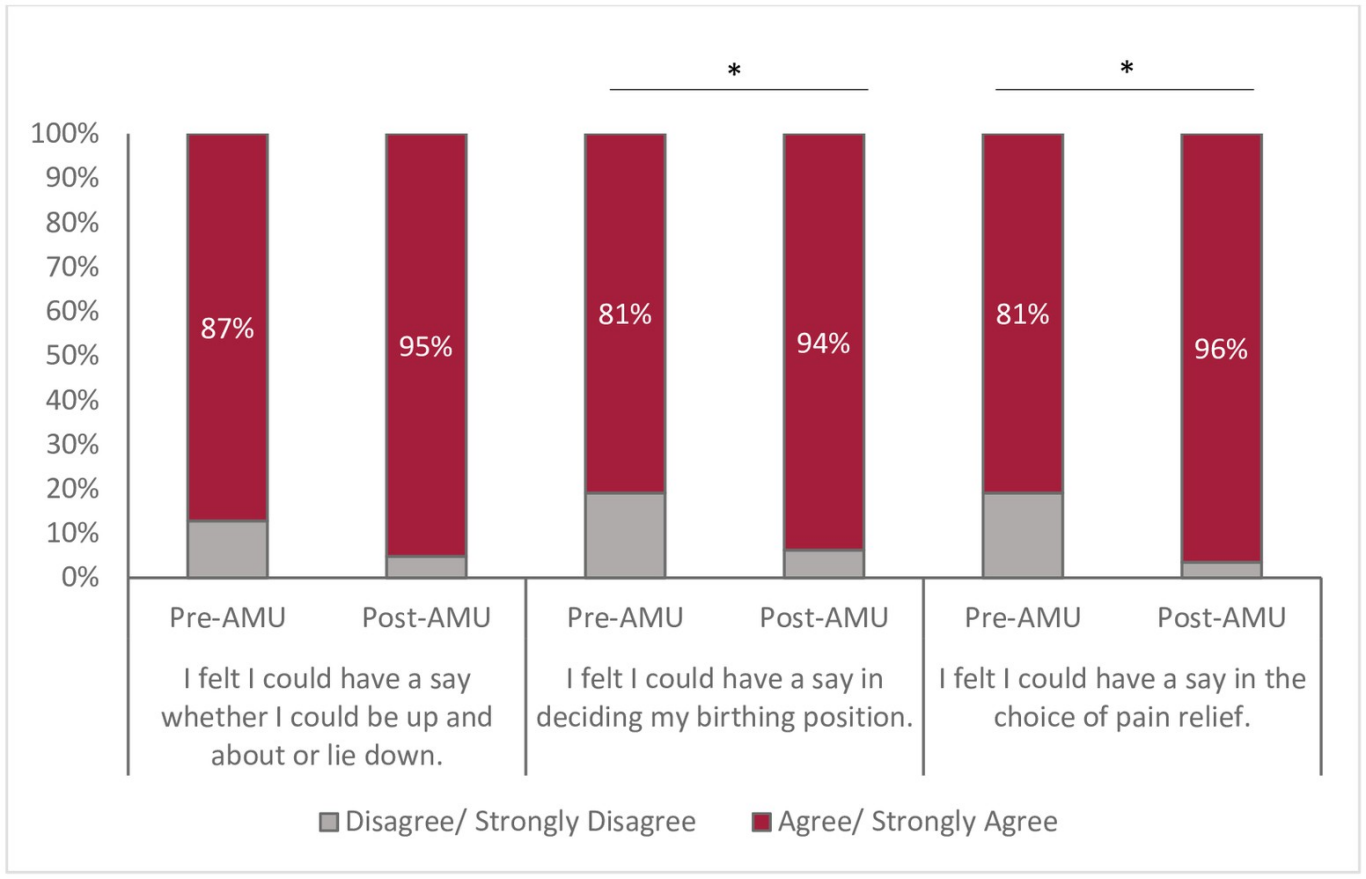
Participants’ perceptions of participation during labour and birth, pre- and post-AMU implementation. Chi-square test (*p<0.05).

Clients felt included in decisions during their labour and birth (Fig 3), with 94% reporting feeling that they could have a say in being mobile or lying down during labour. This sentiment increased over time with 100% of participants feeling included in decisions around mobility 12–18 months after the implementation of the AMU (p = 0.03). Similarly, clients felt they could contribute to decision making about birthing positions (81%) and choice of pain relief (80.9%) but this was significantly improved after the implementation of the AMU (94%, 95% CI 0.89,0.97, p = 0.008, and 97%, 95% CI 0.93,0.99, p<0.001, respectively).

### Perceived safety, control & security

Overall, 90% of participants reported having many positive memories of birth (Table 2). Although 31.3% of all clients agreed to feeling scared during birth, this sentiment was reported considerably less among post-AMU clients (27% (95% CI 0.20,0.35) vs. 45% (95% CI 0.31,0.59 p = 0.02). Few reported having negative memories from birth (16%) and did not agree (93%) that memories of birth elicited feelings of sadness/depression. Perceived safety among participants was high, with 98% feeling secure in the hands of their care provider team.

**Table 2:** Participants’ perceptions of safety during labour and birth, pre- and post-AMU implementation.

Question:	Pre AMU		Post AMU	
	Disagree/Strongly Disagree	Agree/Strongly Agree	Disagree/Strongly Disagree	Agree/Strongly Agree
I felt scared during labour & birth	55.3%	44.7%	73.1%	26.9%
I have positive memories from birth	14.9%	85.1%	8.9%	91.1%
I have negative memories from birth	76.6%	23.4%	87.0%	13.0%
Some memories make me feel depressed	87.2%	12.8%	94.5%	5.5%
The team’s medical skills made me feel secure	6.4%	93.6%	0%	100%

**Fig 4:**
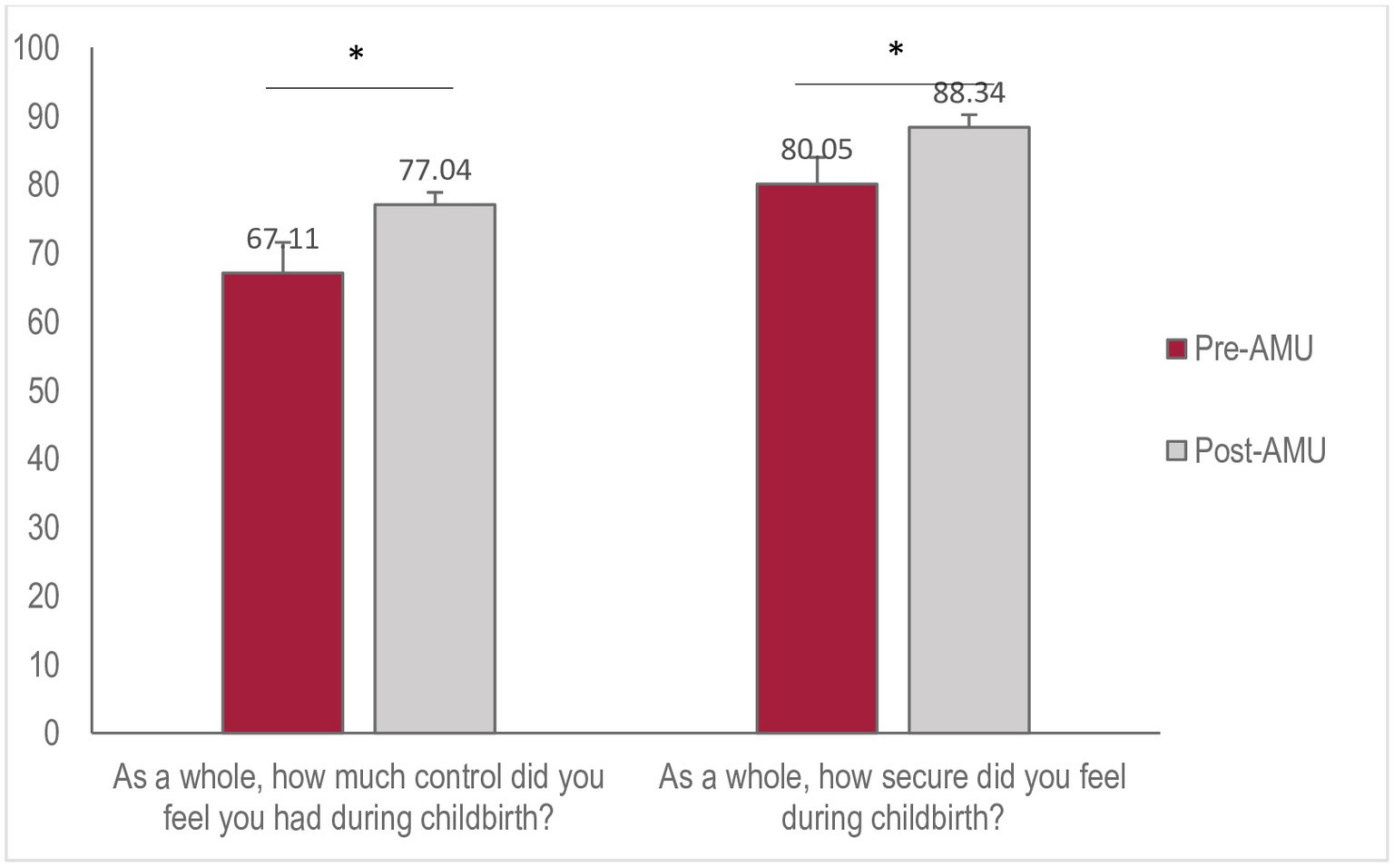
Participants’ perceptions of control and security during labour and birth, pre- and post-AMU implementation. Independent Samples t-test (*p<0.05).

Clients rated their sense of control higher after the AMU opened (67.1±26.53 vs 77.0±.21.97, p = 0.01), and continued to increase one year after its implementation (73.9±.23.55 81.8±.18.51, p = 0.04). When asked to quantify their level of security (using a visual sliding scale, 0 to 100), all clients’ average level of security was 86.4± 16.07 (Fig 4). Feelings of security were higher after the AMU was implemented (88.4±13.82 vs. 80.1±20.71, p = 0.02) and continued to increase 12–18 months after its opening (91.5±11.51 vs 86.3±14.87, p = 0.02).

### Professional support

**Fig 5:**
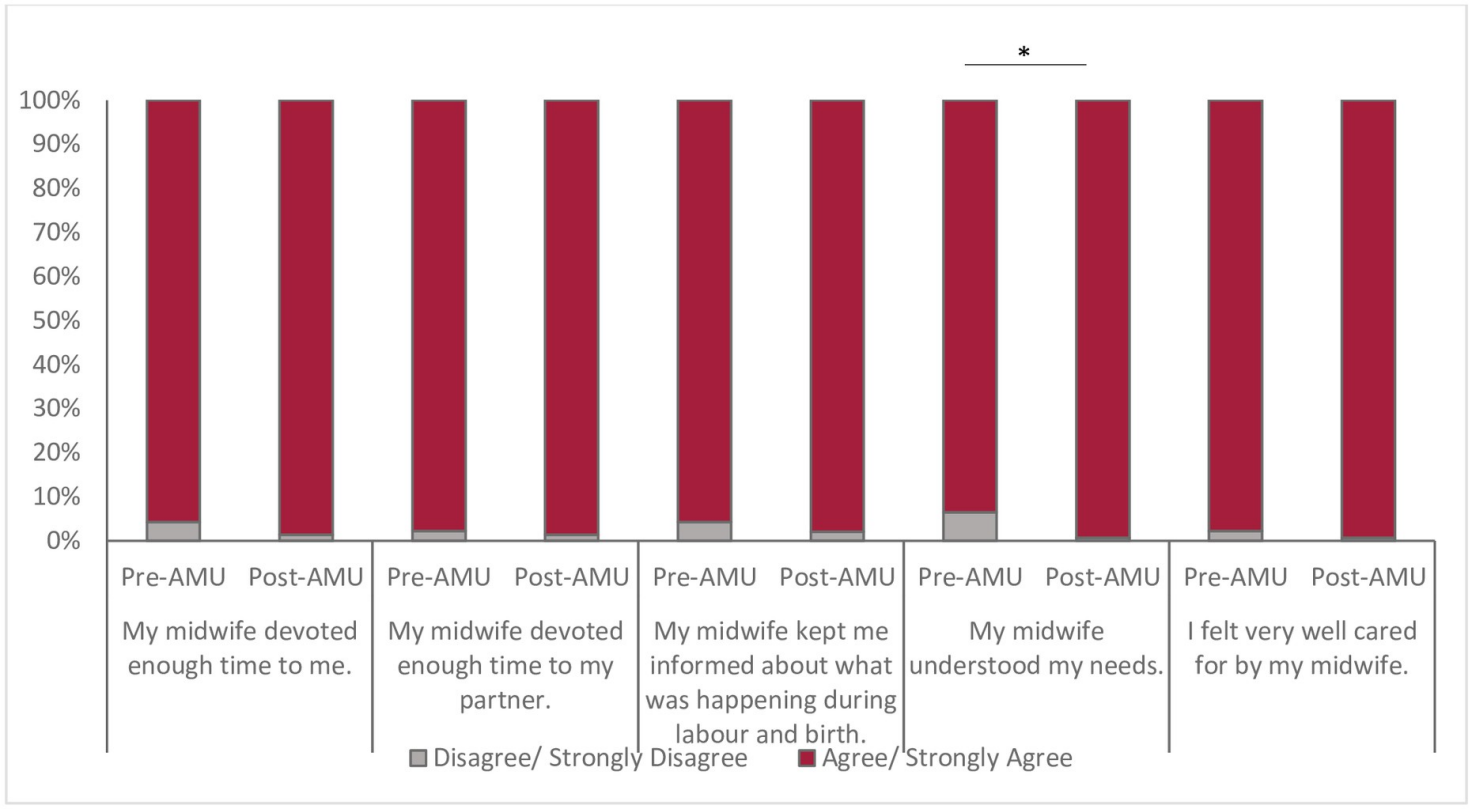
Participants’ perceptions of professional support during labour and birth, pre- and post-AMU implementation. Chi-square test (*p<0.05).

Clients felt very well supported by their midwives throughout their birth experience. Over 97.4% of all clients agreed that their midwife devoted enough time to them and their partner, understood their needs, and kept them informed, and that they were well cared for (Fig 5). More clients who birthed after the AMU was implemented felt their midwife understood their needs (99% (95% CI 0.97,1.0) vs. 94% (95% CI 0.84,0.98, p = 0.02).

### Satisfaction

**Fig 6:**
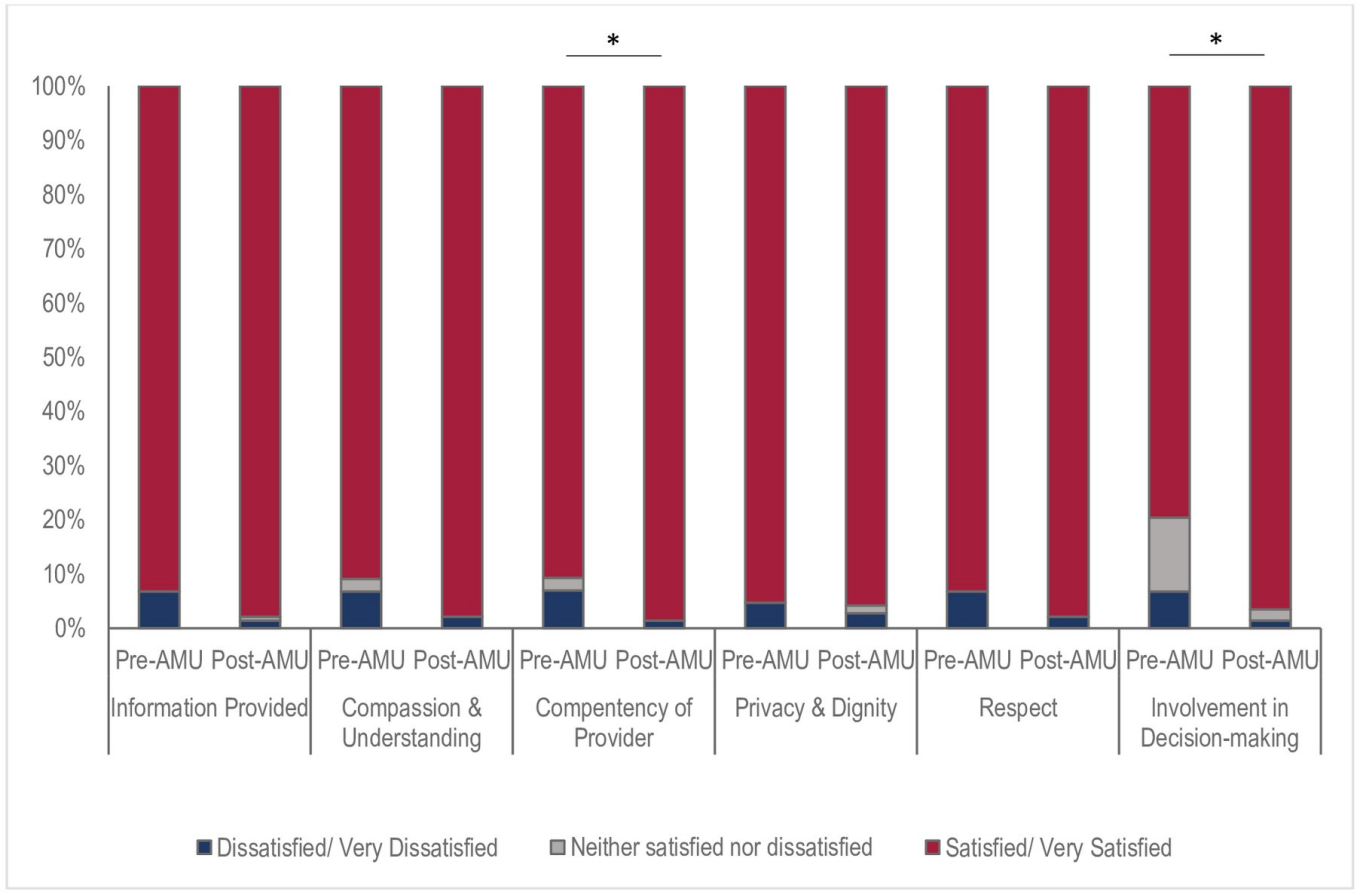
Canadian Maternity Experiences Survey (CMES). Chi-square test (*p<0.05).

All clients reported high levels of satisfaction with their birth experience, irrespective of birth environment (i.e., both before and after the implementation of AMU). Over 96% of all participants were satisfied with the information provided, compassion, privacy and dignity, and respect shown to them during labour and delivery (Fig 6). Clients were satisfied with their involvement during birth and 81% reported feeling very satisfied. Considerably more clients who gave birth after opening of the AMU were happy with their level of involvement during their birth experience (97%–95% CI 0.93,0.99) vs. 80% (95% CI 0.66,0.89, p = 0.001). Likewise, more post-AMU clients reported feeling satisfied with the competency of their midwife (99% (95% CI 0.96,1.0) vs. 91% (95% CI 0.79,0.97, p = 0.03).

## Discussion

The aim of this study was to describe birth experiences and satisfaction on Canada’s first AMU. Our findings indicated that clients had positive experiences in the four domains assessed—participation and capacity; perceived safety, control, and security; professional support; and overall satisfaction; however, there were some differences between groups which indicated higher levels of satisfaction with the AMU model of care.

While levels of midwifery client satisfaction were higher after AMU implementation, pre-AMU clients already had very high rates of satisfaction. This is in keeping with other literature which demonstrates higher rates of satisfaction with midwifery-led care, compared to other care models [21,25,26]. The increase in satisfaction post-implementation suggests that the AMU as an option for birthplace contributes meaningfully to satisfaction, beyond the general satisfaction with the midwifery model. This could be due to a variety of factors, including the new physical environment of the AMU [27], the contributions of the hospitalist midwife, new midwifery specific policies to support both midwives and their clients [28], the unit being self-governed by midwives, and work that occurred to build a positive culture within the midwifery team [28].

Studies have demonstrated the importance of women and childbearing people being active participants in decision making during their birth, regardless of carer [29]. Furthermore, evidence suggests that higher levels of participation results in clients’ perceptions of better birth experiences [30]. Active participation and control over birth experiences are fundamental reasons people choose midwifery care [22,31]. Specifically, midwifery clients seek more options and control over birth and labour positions, pain management, and labour tools. This locus of control has been shown to be a significant predictor of satisfaction during birth with women and birthing people wanting to retain a sense of autonomy and control through active decision-making and participation [32,33]. We hypothesize that the increase in feelings of security and control are likely due, in part, to the new environment of the AMU, which included the physical space and the variety of equipment options available. In the AMU, midwives have more birth tools and equipment to offer their clients than previously, in addition to more spacious rooms, and customizable room features like music and lighting [27]. These features facilitate active participation and greater control over the environment and birth experience.

Professional support was highly rated among participants, likely due to the philosophies underpinning midwifery care itself: informed choice, continuity of care, and choice of birthplace [34]. This was echoed in our in-depth interviews, where clients described how the core elements of the midwifery model of care were maintained irrespective of birth environment. However, more participants in the post-implementation group were satisfied with the competency of their care provider. We theorize this resulted from the midwives being more comfortable and confident on the AMU, since they could take ownership over the space, which was purpose-built for them [27]. Additionally, having the hospitalist midwives on the AMU at all times facilitates midwives’ confidence in working independently [35]. This aligns with the findings of previous components of the AMU evaluation study, which found that healthcare providers perceived that the AMU enhanced the already high-level of quality care provided by midwives [35]. This could similarly explain why fewer clients were scared during birth post-implementation, possibly because they had high levels of confidence in their midwives’ competence, in addition to having an experienced hospitalist midwife to assist.

Continuity of care is a core component of midwifery [36] and a large reason people seek midwifery care is the relationship with the midwife attending the birth [37]. Despite concern that the introduction of the hospitalists midwife might interrupt continuity of care, our survey result corroborate our findings in qualitative interviews that the hospitalists had a positive impact on client satisfaction. We found that rather than disrupting continuity, the AMU supports clients being cared for by a midwife at all times, supports the known midwife to be present at the birth, and allows hospitalists to provide valuable relief and guidance, which may have contributed to post-implementation respondents’ higher levels of confidence in their midwives. Furthermore, the length of data collection suggests that the higher level of satisfaction does not appear to be associated with novelty of the setting but is sustained and improving over time. This may reflect a learning curve for the midwives in becoming more confident in their new setting. Our findings provide reassurance that this innovation of the AMU which modified the already highly successful model of midwifery led care in Ontario did not result in changes that reduced client satisfaction.

Our study is strengthened by the length of data collection, as it ensured that we captured AMU experiences beyond the initial implementation phase. Our findings are potentially limited by selection bias, as clients with particularly good experiences at the AMU may have been more likely to participate. The generalizability of our findings is also limited, as the context of our study is very specific to the Ontario midwifery model of care, and the particular setting of the first AMU; however, our findings may still be transferable to settings with similar models of midwifery and a similar AMU. Due to study limitations, we were not able to compare experiences with obstetric patients over the same time periods or to compare characteristics of our respondents with obstetric patients. This is an opportunity for future research, as it would allow us to draw comparisons between maternity units within the same hospital, rather than simply within the midwifery unit. This study provides a scaffold from which future AMU evaluation studies can be based as more midwife-led units are implemented.

## Conclusion

Giving birth on the AMU is associated with high levels of satisfaction, particularly the perception of being actively engaged in decision making, having a sense of control and safety, and having confidence in the clinical care provider team. When compared to midwifery clients who gave birth on the Labour unit prior to the creation of the AMU, those who gave birth on the AMU had higher levels of satisfaction related to their participation in care and greater sense of security and sense of control, while maintaining the same level of confidence in their care providers. Care received on the AMU does not compromise birth experiences or satisfaction and appears to be associated with greater autonomy and agency for the person giving birth.

## References

[1] Canadian Association of Midwives (CAM). Midwifery across Canada [Internet]. 2020 [cited 2020 Jun 17]. https://canadianmidwives.org/midwifery-across-canada/#1467634074483-f50b550d-db87.

[2] Canadian Association of Midwives (CAM). Midwifery across Canada [Internet]. [cited 2019 Jul 3]. https://canadianmidwives.org/midwifery-across-canada/#1467634074483-f50b550d-db87.

[3] Association of Ontario Midwives (AOM). Midwifery Care [Internet]. 2019. https://www.ontariomidwives.ca/midwifery-care.

[4] Ontario Ministry of Health and Long-Term Care (MoHLTC). What is a Midwife? [Internet]. Midwifery in Ontario. [cited 2021 Mar 9]. http://www.health.gov.on.ca/en/public/programs/midwife/.

[5] Association of Ontario Midwives (AOM). Midwifery by the Numbers [Internet]. 2021 [cited 2021 Aug 13]. https://www.ontariomidwives.ca/midwifery-numbers.

[6] Janssen PA, Ryan EM, Etches DJ, Klein MC, Reime B. Outcomes of planned hospital birth attended by midwives compared with physicians in British Columbia. Birth. 2007;34(2):140–7. doi: 10.1111/j.1523-536X.2007.00160.x 17542818

[7] Hutton EK, Cappeletti A, Reitsma AH, Simioni J, Horne J, McGregor C, et al. Outcomes associated with planned place of birth among women with low-risk pregnancies. CMAJ [Internet]. 2016 [cited 2017 Sep 12];188(5). Available from: https://www.ncbi.nlm.nih.gov/pmc/articles/PMC4786402/pdf/1880e80.pdf. doi: 10.1503/cmaj.150564 26696622 PMC4786402

[8] Mattison CA, Dion ML, Lavis JN, Hutton EK, Wilson MG. Midwifery and obstetrics: Factors influencing mothers’ satisfaction with the birth experience. Birth [Internet]. 2018 Sep 1 [cited 2022 Aug 3];45(3):322–7. Available from: https://onlinelibrary.wiley.com/doi/full/10.1111/birt.12352. 29687481 10.1111/birt.12352

[9] Cameron C, Sassi J, Malott A. The Markham Stouffville Hospital Alongside Midwifery Unit: A centre for excellence for normal birth. Can J Midwifery Res Pr. 2019;19(1):43–52.

[10] Brocklehurst P, Hardy P, Hollowell J, Linsell L, Macfarlane A, McCourt C, et al. Perinatal and maternal outcomes by planned place of birth for healthy women with low risk pregnancies: The Birthplace in England national prospective cohort study. BMJ [Internet]. 2012 Jan 21 [cited 2021 Mar 12];343(7840). Available from: http://www.bmj.com/content/343/bmj.d7400?tab=related#webextra. 10.1136/bmj.d7400 PMC3223531 22117057

[11] Grigg CP, Tracy SK, Tracy M, Daellenbach R, Kensington M, Monk A, et al. Evaluating Maternity Units: A prospective cohort study of freestanding midwife-led primary maternity units in New Zealand—Clinical outcomes. BMJ Open [Internet]. 2017 Aug 1 [cited 2021 Mar 12];7(8):e016288. Available from: http://bmjopen.bmj.com/. doi: 10.1136/bmjopen-2017-016288 28851782 PMC5634452

[12] Borquez HA, Wiegers TA. A comparison of labour and birth experiences of women delivering in a birthing centre and at home in the Netherlands. Midwifery. 2006 Dec 1;22(4):339–47. doi: 10.1016/j.midw.2005.12.004 16647170

[13] Gaudineau A, Sauleau EA, Nisand I, Langer B. Obstetric and neonatal outcomes in a home-like birth centre: A case-control study. Arch Gynecol Obstet. 2013;287(2):211–6. doi: 10.1007/s00404-012-2553-6 22976132

[14] Welffens K, Derisbourg S, Costa E, Englert Y, Pintiaux A, Warnimont M, et al. The “Cocoon,” first alongside midwifery-led unit within a Belgian hospital: Comparison of the maternal and neonatal outcomes with the standard obstetric unit over 2 years. Birth. 2020;47(1):115–22. 31746028 10.1111/birt.12466 PMC7065252

[15] Merz WM, Tascon-Padron L, Puth MT, Heep A, Tietjen SL, Schmid M, et al. Maternal and neonatal outcome of births planned in alongside midwifery units: A cohort study from a tertiary center in Germany. BMC Pregnancy Childbirth [Internet]. 2020 May 6 [cited 2020 Jun 16];20(1):1–10. Available from: https://bmcpregnancychildbirth.biomedcentral.com/articles/10.1186/s12884-020-02962-4. 32375692 10.1186/s12884-020-02962-4 PMC7201515

[16] Maillefer F, de Labrusse C, Cardia-Vonèche L, Hohlfeld P, Stoll B. Women and healthcare providers’ perceptions of a midwife-led unit in a Swiss university hospital: A qualitative study. BMC Pregnancy Childbirth. 2015;15(1):1–11. 25886389 10.1186/s12884-015-0477-4 PMC4359486

[17] Institute for Healthcare Improvement (IHI). The IHI Triple Aim [Internet]. [cited 2021 Mar 12]. http://www.ihi.org/engage/initiatives/tripleaim/Pages/default.aspx.

[18] Sawyer A, Ayers S, Abbott J, Gyte G, Rabe H, Duley L. Measures of satisfaction with care during labour and birth: A comparative review. BMC Pregnancy Childbirth [Internet]. 2013 May 8 [cited 2021 Mar 12];13. Available from: https://pubmed.ncbi.nlm.nih.gov/23656701/. doi: 10.1186/1471-2393-13-108 PMC3659073 23656701

[19] Harvey S, Rach D, Stainton MC, Jarrell J, Brant R. Evaluation of satisfaction with midwifery care. Midwifery. 2002 Dec 1;18(4):260–7. doi: 10.1054/midw.2002.0317 12473441

[20] Goodman P, Mackey MC, Tavakoli AS. Factors related to childbirth satisfaction. J Adv Nurs. 2004 Apr;46(2):212–9. doi: 10.1111/j.1365-2648.2003.02981.x 15056335

[21] Mattison CA, Dion ML, Lavis JN, Hutton EK, Wilson MG. Midwifery and obstetrics: Factors influencing mothers’ satisfaction with the birth experience. Birth [Internet]. 2018 Sep 1 [cited 2020 Mar 9];45(3):322–7. Available from: http://doi.wiley.com/10.1111/birt.12352. 29687481 10.1111/birt.12352

[22] Murray-Davis B, Grenier L, Mattison CA, Malott A, Cameron C, Li J, et al. Mediating expectations and experiences that influence birth experiences in Canada’s first Alongside Midwifery Unit. Birth Issues Perinat Care. 2022;TBD(TBD):TBD. 10.1111/birt.12744 37485759

[23] Dencker A, Taft C, Bergqvist L, Lilja H, Berg M. Childbirth experience questionnaire (CEQ): development and evaluation of a multidimensional instrument. BMC Pregnancy Childbirth [Internet]. 2010 Dec 10 [cited 2019 Jun 20];10(1):81. Available from: https://bmcpregnancychildbirth.biomedcentral.com/articles/10.1186/1471-2393-10-81. 21143961 10.1186/1471-2393-10-81 PMC3008689

[24] Public Health Agency of Canada (PHAC). What Mothers Say: The Canadian Maternity Experiences Survey [Internet]. 2009 [cited 2019 Jun 20]. http://www.publichealth.gc.ca/mes.

[25] Bernitz S, Øian P, Sandvik L, Blix E. Evaluation of satisfaction with care in a midwifery unit and an obstetric unit: a randomized controlled trial of low-risk women. BMC Pregnancy Childbirth [Internet]. 2016 Dec 18 [cited 2019 Sep 9];16(1):143. Available from: http://bmcpregnancychildbirth.biomedcentral.com/articles/10.1186/s12884-016-0932-x. 27316335 10.1186/s12884-016-0932-x PMC4912783

[26] Sandall J, Soltani H, Gates S, Shennan A, Devane D. Midwife-led continuity models versus other models of care for childbearing women. Cochrane Database Syst Rev [Internet]. 2016 Apr 28 [cited 2019 Sep 10];4(4). Available from: http://doi.wiley.com/10.1002/14651858.CD004667.pub5. 27121907 10.1002/14651858.CD004667.pub5 PMC8663203

[27] Murray-Davis B, Grenier LN, Plett RA, Mattison CA, Ahmed M, Malott AM, et al. Making Space for Midwifery in a Hospital: Exploring the Built Birth Environment of Canada’s First Alongside Midwifery Unit. Heal Environ Res Des J. 2022;1–19. 10.1177/19375867221137099 PMC10133785 36384318

[28] Darling EK, Easterbrook R, Grenier LN, Malott A, Murray-Davis B, Mattison CA. Lessons learned from the implementation of Canada’s first alongside midwifery unit: A qualitative explanatory study. Midwifery. 2021 Dec 1;103:103146. doi: 10.1016/j.midw.2021.103146 34592575

[29] Fawsitt CG, Bourke J, Lutomski JE, Meaney S, McElroy B, Murphy R, et al. What women want: Exploring pregnant women’s preferences for alternative models of maternity care. Health Policy (New York) [Internet]. 2017;121(1):66–74. Available from: 10.1016/j.healthpol.2016.10.010. 27884492

[30] Sjödin M, Rådestad I, Zwedberg S. A qualitative study showing women’s participation and empowerment in instrumental vaginal births. Women and Birth. 2018;31(3):185–9. doi: 10.1016/j.wombi.2017.09.006 28943318

[31] Davison C, Hauck YL, Bayes SJ, Kuliukas LJ, Wood J. The relationship is everything: Women’s reasons for choosing a privately practising midwife in Western Australia. Midwifery [Internet]. 2015 Aug 1 [cited 2020 Aug 13];31(8):772–8. Available from: https://pubmed.ncbi.nlm.nih.gov/26001949/. 26001949 10.1016/j.midw.2015.04.012

[32] Fair CD, Morrison TE. The relationship between prenatal control, expectations, experienced control, and birth satisfaction among primiparous women. Midwifery. 2012;28(1):39–44. doi: 10.1016/j.midw.2010.10.013 21458895

[33] Downe S, Finlayson K, Oladapo O, Bonet M, Gülmezoglu AM. What matters to women during childbirth: A systematic qualitative review. PLoS One [Internet]. 2018 Apr 1 [cited 2022 Aug 4];13(4):e0194906. Available from: https://journals.plos.org/plosone/article?id=10.1371/journal.pone.0194906. 29664907 10.1371/journal.pone.0194906 PMC5903648

[34] Association of Ontario Midwives (AOM). Midwifery Care [Internet]. About Midwifery. 2021 [cited 2021 Mar 9]. https://www.ontariomidwives.ca/midwifery-care.

[35] Murray-Davis B, Grenier LN, Mattison CA, Malott A, Cameron C, Hutton EK, et al. Promoting safety and role clarity among health professionals on Canada’s First Alongside Midwifery Unit (AMU): A mixed-methods evaluation. Midwifery [Internet]. 2022 Aug 1 [cited 2022 Aug 3];111:103366. Available from: https://linkinghub.elsevier.com/retrieve/pii/S0266613822001188. doi: 10.1016/j.midw.2022.103366 35594803

[36] Rocca-Ihenacho L, Yuill C, McCourt C. Relationships and trust: Two key pillars of a well-functioning freestanding midwifery unit. Birth. 2021 Mar 1;48(1):104–13. doi: 10.1111/birt.12521 33314346

[37] Perriman N, Davis DL, Ferguson S. What women value in the midwifery continuity of care model: A systematic review with meta-synthesis. Midwifery [Internet]. 2018;62(February):220–9. Available from: doi: 10.1016/j.midw.2018.04.011 29723790

